# Research with undocumented migrant participants

**DOI:** 10.15649/cuidarte.4621

**Published:** 2024-12-19

**Authors:** Daniella Cancino-Jiménez, René Leyva Flores, Miguel Valencia-Contrera, Jorge Cancino-Jiménez, Naldy Febré

**Affiliations:** 1 Universidad Andres Bello, Facultad de Enfermería. Santiago, Chile. E-mail: daniella.cancino@unab.cl Universidad Andres Bello Universidad Andres Bello Santiago Chile daniella.cancino@unab.cl; 2 Center for Research in Health Systems (CISS), National Institute of Public Health (INSP), Morelos, México. E-mail: rene.leyva@insp.mx Center for Research in Health Systems National Institute of Public Health Morelos México rene.leyva@insp.mx; 3 Universidad Andres Bello, Facultad de Enfermería. Santiago, Chile. E-mail: miguel.valencia@unab.cl Universidad Andres Bello Universidad Andres Bello Santiago Chile miguel.valencia@unab.cl; 4 Universidad de Santiago, Facultad de Ciencias Médicas. Santiago, Chile. E-mail: jorge.cancino@usach.cl Universidad de Santiago Universidad de Santiago Santiago Chile jorge.cancino@usach.cl; 5 Universidad Andres Bello, Facultad de Enfermería. Santiago, Chile. E-mail: naldy.febre@unab.cl Universidad Andres Bello Universidad Andres Bello Santiago Chile naldy.febre@unab.cl

Globally, migration is increasing at an accelerated and sustained rate, driven by social, political, and economic factors that prompt the displacement of people across countries, resulting in significant migratory waves^1^–^4^. It is estimated that there are 272 million international migrants worldwide^3^. Migration is defined by the International Organization for Migration (IOM) as the “Movement of persons away from their place of usual residence, either across an international border or within a State”^5^. 

In the specific case of the American continent, the situation of the Venezuelan population is particularly noteworthy today. As the country's situation persists or fails to improve over time, massive returns have not occurred. Instead, the economic and social integration processes of Venezuelan migrants in their host countries are strengthened^6^. 

From a health perspective, migration is recognized as a social determinant of health (SDH), as it brings about important lifestyle changes associated with the migration process itself (pre-, during, and post-migration phases)^7^. It often intersects and conflicts with other SDHs, such as access to healthcare, education, social status, and working conditions. Consequently, the migrant population faces heightened physical and mental health risks, along with increased healthcare inequalities (reduced access, coercion, among other challenges). Additionally, migrants encounter barriers such as issues with acceptability and dissatisfaction with health communication, which heighten their vulnerability by discouraging the use of health services due to discriminatory treatment and racism^8^–^15^. 

Within this already vulnerable population, there is a subgroup that experiences an exacerbation of the challenges described above: 'irregular or undocumented migrants.' Their situation is further compounded by additional barriers, including the need to remain permanently in the host country, fear of fines, violence, imprisonment, deportation, and stigmatization^15^,^16^. 

In this context, which has significant implications for public health, continuing and expanding existing research efforts is imperative. These efforts form the scientific foundation necessary to develop and deliver health responses that are coherent, effective, and efficient, addressing the new and diverse needs arising from sociodemographic, political, and epidemiological changes. Such responses must also be relevant and culturally appropriate to the evolving characteristics and population compositions across different regions of the world. It is essential to understand the needs of this population group. 

In this line, within research contexts, upholding and adhering to ethical principles that protect this population is essential. This includes addressing the challenges of collecting information due to fears of being reported, the heightened vulnerability associated with the migratory process, and the complex political, social, economic, and health contexts in which they find themselves. 

While international ethical standards aim to ensure the respect and protection of all research participants, studies involving migrants—who often live in a state of continuous social suffering—require additional safeguards. It is necessary to monitor the information generated, the methodological procedures employed, and the approaches and interactions used^17^. 

In this context, the 2016 international ethical guidelines for health-related research involving human subjects provided recommendations for the special protection of potentially vulnerable individuals. These include ensuring that procedures posing no potential individual benefit to participants involve no more than minimal risk and requiring that research be conducted only when it addresses conditions specifically affecting these groups^18^. 

Given that research is an intentional process, it is essential to establish ethical guidelines or frameworks to be followed at every stage and ensure the protection of participants' dignity, integrity, and safety. In this sense, in matters of migration, it is unavoidable to complement these general ethical frameworks with specific considerations, accounting for the social nature of the migratory phenomenon, the theoretical advances in the field^19^, human rights, the cultural characteristics of the participants^20^, and their life histories, among other factors. 

From this perspective, it is crucial to prevent symbolic violence, which can manifest through asymmetrical relationships between researchers and study participants, the imposition of the researcher's own conceptual frameworks, and hierarchical dynamics of superiority and inferiority^19^. Similarly, in intercultural or global research, a pluriversal ethical framework is necessary, aiming to strike a balance between respecting human rights and cultural diversity^20^. 

A notable aspect now acknowledged is that 'migration is a total social fact that makes it particularly complex to develop a set of ethical principles to guide research practices,'^19^ given the countless multicultural characteristics and the immense diversity of contexts on a global scale^20^0. 

However, as a society, addressing these aspects with a central focus on the human dimension becomes a crucial driving force for promoting social integration and inclusion. In this regard, respect for human rights is seen as a tool for promoting the fulfillment of basic developmental needs, non-discrimination, and physical and psychological integrity. It is characterized by its universality and inclusivity, emphasizing elements such as integrality, interdependence, collectivity, and equity^21^. 

Finally, the participation of undocumented migrants in research is a controversial issue that raises several ethical concerns. Interacting with research teams lacking the necessary skills and sensitivity further exacerbates the social suffering they endure. Consequently, there is an urgent need to find guidance tools on how to protect this vulnerable population. 

Iterative reflection aimed at balancing cultural diversity, respect for international conventions or agreements, and the legislation in force in the countries where research is conducted would facilitate the adoption of ethical approaches to studies involving migrant participants. This process would enable the making of ethically sound and justified decisions^22^. 

Given the nature of the irregular migration phenomenon, risks and vulnerabilities coexist, demanding special attention to give a coherent response that safeguards security and confidentiality throughout the research process in contexts where participants could be disadvantaged by the production of information, exposing them to psychological, physical, or legal risks. 

Nursing science is particularly relevant in this area. Although it faces limitations and challenges, it offers unique conditions, competencies, and a well-developed theoretical foundation. Grounded in its metaparadigm—which considers the interaction between the person, the environment or society, health, and nursing—nursing science provides models and theories that guide both clinical practice and research. Thus, the ethical knowledge inherent to the discipline helps clarify conflicts and facilitates the exploration of alternatives based on principles and values^23^ as part of critical thinking and nursing actions.
